# Resource Centers for Minority Aging Research (RCMAR) Fostering Community Engagement

**DOI:** 10.1093/geroni/igaf122.1414

**Published:** 2025-12-31

**Authors:** David Lee, Yaolin Pei

## Abstract

The Resource Centers for Minority Aging Research (RCMAR) Program has been continuously funded by the National Institute on Aging (NIA) since 1997. The RCMAR Program has two primary objectives: to support the career development of early-career scientists conducting social, behavioral, psychological, and economic research related to aging, health disparities in older adults, and/or Alzheimer’s Disease and Alzheimer’s Disease-related dementias (AD/ADRD), and to advance scientific discoveries related to aging. This includes efforts to eliminate health disparities and inequities and to improve the health and well-being of older adults. There are 18 RCMAR Centers in the United States, 10 are focused on AD/ADRD and 8 are focused on aging.

